# Hospital pharmacists’ perceptions of the suitability of doctor of pharmacy graduates in hospital settings in Thailand

**DOI:** 10.1186/s12909-015-0471-6

**Published:** 2015-10-24

**Authors:** Teeraporn Chanakit, Bee Yean Low, Payom Wongpoowarak, Summana Moolasarn, Claire Anderson

**Affiliations:** 1 School of Pharmacy, University of Nottingham, Nottingham, NG7 2RD UK; 2 School of Pharmacy, Faculty of Science, University of Nottingham Malaysia Campus, Jalan Broga, 43500 Semenyih, Selangor Darul Ehsan Malaysia; 3 Faculty of Pharmaceutical Sciences, Prince of Songkla University, Hat Yai, Songkhla 90112 Thailand; 4 Faculty of Pharmaceutical Sciences, Ubon Ratchathani University, Warin Chamrap, Ubon Ratchathani 34190 Thailand

## Abstract

**Background:**

Thai pharmacy education has moved to an all Doctor of Pharmacy (PharmD) programme. However, there has been no previous research about the perceptions regarding the suitability of PharmD graduates employed in hospital settings, which is the major pharmacy workforce in Thailand.

**Methods:**

A cross-sectional survey questionnaire was distributed to 180 hospital pharmacists at the 2013 Association of Hospital Pharmacy (Thailand) conference. This study aimed to explore Thai hospital pharmacists’ perceptions concerning the suitability of the PharmD graduates employed in hospital settings and the competency differences between the Bachelor of Pharmacy (BPharm) and PharmD graduates. Descriptive statistics were used to present the participants’ demographics and their perceptions. An inductive thematic analysis was used to analyse the open-ended written answers.

**Results:**

Ninety-eight valid responses were included in the data analysis (response rate of 55.6 %). The majority of the respondents (76.5 %) felt that the PharmD graduates were suited for the hospital setting and addressed its need for more professionals working in pharmaceutical care and with multi-disciplinary teams. Approximately 55 % of respondents agreed that there were competency differences between the BPharm and PharmD graduates. Major themes emerged in response to the open-ended written answers showing that PharmD graduates had high competency in patient care services and readiness to work, particularly in large hospitals, due to their training to work in specialised areas (e.g., special clinics, ward rounds). However, PharmD graduates require more training in health promotion and humanistic skills and need the system to promote the role of PharmD in pharmaceutical care.

**Conclusions:**

PharmD graduates were suited for hospital settings. However, there were concerns regarding the suitability of the PharmD graduates for the community hospital and primary care hospital settings because of their insufficient training in health promotion and disease prevention. Half of the respondents perceived PharmD graduates as having higher competencies in clinical activities and being more prepared to work than BPharm graduates. However, the other half of the respondents perceived the competency of both pharmacy qualifications as being similar, as PharmD graduates provide non-clinical activities similar to BPharm graduates due to the high workload in dispensing services and the shortage of hospital pharmacists, which prevent PharmD graduates from providing direct pharmaceutical care services.

**Electronic supplementary material:**

The online version of this article (doi:10.1186/s12909-015-0471-6) contains supplementary material, which is available to authorized users.

## Background

Thai pharmacists are required to become involved in all aspects of the pharmaceutical supply chain, from the pharmaceutical industry to monitoring patient outcomes [1]. The sectors in which Thai pharmacists work are categorised as hospitals (40 %), pharmacy marketing (22 %), community pharmacies (17 %), the pharmaceutical industry (10 %), consumer protection (6 %) and education (5 %) [2, 3].

Hospital pharmacists form a major part of the pharmacy workforce in Thailand [3]. A survey about hospital pharmacist qualifications by the Association of Hospital Pharmacy (Thailand) in 2009 showed that only 7 % of hospital pharmacists were 6-year Doctor of Pharmacy (PharmD) graduates, whereas the majority of hospital pharmacists were 5-year Bachelor of Pharmacy (BPharm) graduates (72 %) and the remaining were postgraduates (e.g., master’s degree, doctor of philosophy (PhD) and board-certified specialists) (21 %) [4]. Their major functions are dispensing, compounding, procurement and clinical pharmacy activities. The majority of hospital pharmacy services in public and private hospitals are out-patient services (public: 99.8 %, private: 100 %), in-patient services (98.5 %, 99.3 %), and drug procurement (97.1 %, 82.6 %). Public and private hospitals include clinical pharmacy-related activities provided by pharmacy graduates, including ambulatory care (67.2 %, 34.8 %), acute care (37.3 %, 15.9 %), drug information services (72.1 %, 52.2 %), medication reconciliation (45.2 %, 29 %), IV admixture preparation (4.9 %, 0 %) and extemporaneous preparation (47 %, 36.2 %) [4].

Hospital pharmacists must participate more in clinical roles (e.g., drug use decision-making, selection of drug products, determination of the dose and dosage schedule, preparation of drug product for patient use, monitoring of drug use, and provision of drug information to patients) which require pharmacy graduates who have more expertise in pharmaceutical care [5]. Changes in the pharmacy curriculum were required to meet these responsibilities [1].

The first pharmacy school in Thailand was started in 1914. It was a 3-year programme and expanded to a 4-year programme in 1941 and then to a 5-year BPharm programme in 1957. The 5-year BPharm programme focused on a diversity of subjects, aiming to produce pharmacists who worked in numerous practices, such as hospitals, community pharmacies, and pharmaceutical industry settings [1, 6].

In 1989, the concept of clinical pharmacy was introduced in Thailand. There was a great demand for pharmacy services in patient care areas [6]. In 1993, the US-Thai Consortium for the Development of Pharmacy Education in Thailand was established [7]. Thai pharmacy educators and pharmacy practitioners participated in the collaboration, which was the motivation for pharmacy educators to develop the PharmD programmes. The first 6-year PharmD programme or the traditional 6-year PharmD programme that focused on patient care only as in the USA was first developed at the Faculty of Pharmaceutical Sciences, Naresuan University in 1999 [1, 8]. This programme aimed to increase the readiness of students to fulfil the needs of society relating to higher competency in patient care.

From 1990–2010, the majority of the universities offered a 5-year BPharm programme that included a number of different tracks, such as pharmaceutical technology, pharmaceutical care, and social and administrative pharmacy.

However, after the Pharmacy Council of Thailand (PCT) announcement in 2008 that only the graduates of the 6-year PharmD programme that complied with the PCT competency standard would be eligible for pharmacy licensure starting from year 2014 onwards [1, 9–11], the 5-year BPharm programme was not offered after 2010.

Policy makers believed that the 6-year programme would move pharmacy competencies from generalist to specialist, resolve the curriculum overload and provide a national standard within the pharmacy profession in Thailand [11]. Additional file 1 shows differences among the curriculum structures between the 5-year BPharm programme, the 2008 Announced 6-year PharmD programme, which focused on patient care, and the 2012 Announced 6-year PharmD programme [9, 12, 13]. Before 2015, the pharmacy graduates included only 5-year BPharm graduates and the 2008 Announced 6-year PharmD programme graduates, and there were no pharmacy graduates from the 2012 Announced 6-year PharmD programme. However, all pharmacy qualifications in Thailand give the pharmacy graduates the same license to work across all sectors.

The US PharmD curriculum and the 2008 Announced 6-year PharmD programme in Thailand focused on patient care. However, the 2012 Announced 6-year PharmD programme in Thailand was adapted to meet the country’s needs resulting from the fact that Thai pharmacy services were still involved in both pharmaceutical care and industrial pharmacy areas. Therefore, the 2012 Announced 6-year PharmD programme in Thailand was divided into two main tracks: the Pharmaceutical Care-PharmD programme (PC-PD) and the Industrial Pharmacy-PharmD programme (IP-PD) [6, 11].

The PCT established competency standards in 2002, which were used as a foundation for the pharmacy curricula and licensure examination and as guidelines for the standard practice of Thai pharmacists. Thai pharmacy graduates from the 5-year BPharm and traditional 6-year PharmD programme and the 2008 Announced 6-year PharmD programme must take the same pharmacy licensure examination based on the 2002 Thai competency standards. These Thai pharmacy competency standards contained 8 domains [7].

After the announcement of the 2012 Announced 6-year PharmD programme, the PCT developed “core competency standards” in 2012 for the 2012 Announced 6-year PharmD programme, which contained 7 domains. The PCT also develop “the functional competency standards in pharmaceutical care” in 2011 (containing 9 domains) and “the functional competency standards in industrial pharmacy” in 2014 (containing 4 domains). Pharmacy students who start their PharmD programme in and after the year 2015 will have to follow the new competency guidelines (e.g., the core competency guidelines and the functional competency standards, which depend on their area of expertise) and have to take the national license examination of core competency standard after completion of the 4^th^ year and the functional competency standards either on pharmaceutical care or industrial pharmacy after completion of the 6^th^ year. Additional file 2 compares the competency standard guidelines for licensure examination of the 5-year BPharm, the 2008 Announced 6-year PharmD programme and the 2012 Announced 6-year PharmD programme [14–17].

**Fig. 1:**
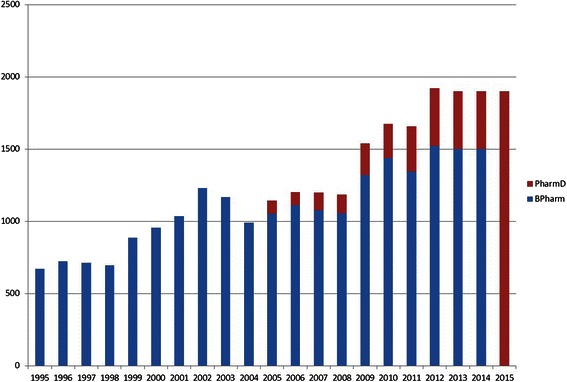
Number of BPharm graduates and PharmD graduates The number of the Thai BPharm and PharmD graduates from 1995–2015. In 2013, the number of the Thai PharmD graduates was only 1,600 (8 % of all 20,000 pharmacy graduates in Thailand). During 2005–2014, there were PharmD graduates from PharmD in pharmaceutical care programme only. In 2015, there are PharmD graduates from the 2012 Announced 6-year PharmD programme

From 2005–2013, the total number of the Thai PharmD graduates from the 6-year PharmD programme was only 1,600 (approximately 8 % of the total existing 20,000 registered pharmacists in Thailand) [3, 18] (see Fig. 1). However, since the PCT’s announcement that only the 6-year programme would be eligible for the licensure examination, all 19 Thai pharmacy schools have changed their curriculum to the 6-year PharmD programme since 2010. Therefore, new pharmacy graduates will be entirely PharmD graduates starting in 2015.

To the best of our knowledge, only one study, by Sitaruno et al., has been completed on satisfaction regarding the skills of Thai PharmD graduates from one university in providing pharmaceutical care based on the opinions of the graduates themselves, employers, colleagues and patients [19]. The study reported on the number of Thai PharmD graduates who provided primary clinical activities (*n* = 34) in hospital settings as follows: pharmaceutical care (38.2 %), ambulatory care (20.6 %), and acute care/ward rounds (14.7 %). The characteristics of PharmD graduates that the employers rated most satisfying were high responsibility and self-learning competencies, whereas PharmD graduates’ colleagues were satisfied with all characteristics of PharmD graduates (e.g., communication skills, leadership, service mind, and self-confidence) [19]. Another study described self-reported professional competency by comparing Pharmaceutical Care track-BPharm (PC-BPharm) and Pharmaceutical Sciences track-BPharm (PSc-BPharm) and traditional 6-year PharmD graduates enrolled in the public service programme [6]. The findings showed that PharmD graduates reported the highest competency in acute care, medication reconciliation and primary care services, whereas PSc-BPharm graduates reported more competence in consumer health protection than PharmD and PC-BPharm graduates [6].

However, there are no studies regarding hospital pharmacists’ perceptions of pharmacy graduates with regard to an overview of the professional suitability for employment in hospital settings. Therefore, this study aimed to explore the perceptions of Thai hospital pharmacists regarding the suitability of PharmD graduates employed in hospital settings in Thailand and their perceptions of the competency differences between BPharm and PharmD graduates. This study might be of interest to employers in hospital pharmacy settings as well as to human resources groups who are involved in employment of the pharmacy workforce. This study may also be helpful for academic institutions and regulatory bodies for reviewing curricula and effectively preparing PharmD graduates to meet the needs of the specific health care system in Thailand and for developing national competency standards for the pharmacy licensure examination.

## Methods

This study forms part of a larger study which aims to explore the perceptions of Thai hospital pharmacists towards an overview of pharmacy education in Thailand. However, this study mainly focused on the hospital pharmacists’ perceptions of the suitability of PharmD graduates employed in hospital settings and the competency differences between BPharm and PharmD graduates. A cross-sectional survey questionnaire was distributed to hospital pharmacists at the Association of Hospital Pharmacy conference in 2013. Suitability and competency were defined as follows:

1. Suitability is the condition of being fitted for or appropriate to a purpose [20]. Pharmacy professional suitability means the fitness of PharmD graduates to practice in that they are able to demonstrate an agreed-upon pharmacy professional standard, professional knowledge, skills and appropriate behaviour to perform pharmacy services safely and effectively in hospital settings. The assessment of professional suitability is important for benefit to the public from pharmacy practices, for the reputation of the pharmacy programme and for the reputation of the pharmacy profession [21–24].
2. Competency refers to PharmD graduates’ capacity to perform actions in hospital workplace settings as perceived by hospital pharmacists for the competency standard guidelines required for both BPharm and PharmD graduates for the licensure examination during the 2002–2014 period [14].

The respondents were Thai hospital pharmacists who participated in this conference. Those who agreed to take part were requested to return the completed questionnaire with written informed consent to the researchers’ team at the end of the conference. Attendees were excluded if they were not a hospital pharmacist.

The questionnaire included two types of questions: 1) closed-ended questions by using dichotomous questions (yes/no) and 2) open-ended questions that aimed to explore the meaning of the responses. The questionnaire consisted of two sections. Section One collected data pertaining to pharmacists’ demographics, whereas Section Two collected data related to respondents’ perceptions of the suitability of the PharmD graduates to work in hospital settings and the competency differences between BPharm and PharmD graduates.

The first draft of the questionnaire was tested for content validity by three Thai experts (two from hospital pharmacy practices and one from the Faculty of Pharmaceutical Sciences in Thailand). All items of the questionnaire had an Item-Objective Congruence (IOC) index score higher than 0.5 [25], which confirmed the content validity of the questionnaire. Based on the recommendations of the experts, a second draft of the questionnaire was constructed, separating items for enhanced clarity and ease of comprehension. A pilot study was undertaken via a convenience sampling of twenty hospital pharmacists via e-mail. The pre-test Cronbach’s alpha reliability was 0.85. The questionnaire took approximately 10 minutes to complete. However, some respondents did not have experience working with PharmD graduates due to the small number of PharmD graduates working in hospitals in 2013 (only approximately 7 % of hospital pharmacists). Although they did not have experience working with PharmD graduates, they were familiar with the roles, responsibilities and competencies of PharmD graduates because they were preceptors for PharmD graduates or had clerkship experiences with PharmD graduates when they were pharmacy students.

An ideal sample would include all hospital pharmacists. However, there was an absence of an up-to-date database representing the numbers and addresses of hospital pharmacists [3, 26]. Therefore, this study used convenience sampling which had the advantages of saving time and cost. However the disadvantage is not reliable for making inferences to the population [27].

The researchers discussed the inclusion criteria that all Thai hospital pharmacists who participated in this conference should be included, regardless of whether PharmD graduates were already working within their hospitals, to explore the current perceptions of hospital pharmacists who had or did not have experience working with PharmD graduates. Respondents who were lacking experience working with PharmD graduates may answer the questionnaire from their attitudes rather than their experiences, which influence their perceptions and might affect the reliability of comments about the suitability and competencies of PharmD graduates. Minor wording changes in some sentences were made after the pilot study, and the questionnaire was modified to the final version.

The data were coded and entered into IBM SPSS software (SPSS for Windows, version 21.0, SPSS Inc., Chicago, IL, USA). We cleaned ten percent of respondents’ data by double-checking to ensure that the data were entered accurately and completely. Ten percent of respondents’ data were randomised by using Microsoft Excel software. Ten records were checked, and one error was found. This error pertained to the highest academic degree. The respondent had two academic degrees (a 5-year BPharm and a master’s degree), but it was entered as only a 5-year BPharm degree. After rechecking, it was amended from only a 5-year BPharm to include a master’s degree.

Descriptive statistics (frequencies and percentages) were used to describe the participants’ demographics and their perceptions. Pearson Chi-square tests were performed to determine the association between the variables, and Fisher’s exact tests were used when more than 20 % of the cells had an expected count of less than 5 [28]. Statistical significance was accepted at a two-tailed p-value of less than 0.05 [29].

An inductive thematic analysis (concepts generated from the qualitative data) [30] was used to analyse the open-ended written answers. The collected information was in the Thai language. The responses were translated from Thai to English for data analysis because this study is a PhD project in the UK and an English transcript had to be prepared to comply with the required audit trail [31] by the Thai and non-Thai researchers. The audit trail was an essential part of rigorous qualitative study, e.g., tracking how the data were analysed and interpreted to assess the trustworthiness of the study [32]. Details of the forward-backward translation [33, 34] are provided below:

1. Two bilingual Thai-English pharmacy academic researchers performed the forward translation of the Thai text transcripts (source language) into English (target language) independently. The two researchers (TC, PY) reached a consensus regarding the English translation.
2. A blind back-translation from English into Thai was undertaken by a fluent Thai-English bilingual speaker for 25 % of the transcripts.
3. Two Thai versions were compared. Researchers confirmed the consistency between the Thai and English versions.

The coding process assigned codes to data segments by using NVivo qualitative data analysis software (QSR International Pty Ltd., Version 10, 2012). An inductive thematic analysis was repeated by two researchers to ensure reliability or trustworthiness; documentation of the steps in the process of analysis makes it transparent for other researchers to review and enables procedures to be accurately repeated if required. Triangulation of data from different sources (e.g., open-ended written answers, government documents) and using verbatim quotes of what participants actually written connected the researchers’ interpretations to increase the validity of the research findings [31, 32, 35].

This study was conducted in accordance with the ethical approved protocol which obtained from the Faculty of Science, University of Nottingham, United Kingdom and Ubon Ratchathani University, Thailand. Participation was voluntary and participants gave written inform consent returned with the completed questionnaire.

## Results

Questionnaires were distributed to 180 conference attendees. Completed surveys were received from 100 respondents, yielding a response rate of 55.6 %. Two respondents were excluded because they were not hospital pharmacists. Therefore, 98 valid responses were included in the data analysis. The characteristics of the respondents are presented in Table 1. The majority of the respondents (*n* = 75, 76.5 %) agreed that the PharmD graduates were suitable for working in hospital settings. Approximately half of the respondents (*n* = 54, 55.1 %) agreed that there were competency differences between the BPharm and PharmD graduates (Table 2).

**Table 1:** Respondents’ characteristics (*n* = 98)

Characteristics	Frequency	Percentage
Gender		
Male	26	26.5
Female	72	73.5
Age range		
<25 years	5	5.1
25–35 years	59	60.2
36–45 years	28	28.6
46–55 years	6	6.1
Highest academic degree		
BPharm^a^ no track	40	40.8
BPharm, track Pharmaceutical care	17	17.3
BPharm, track Pharmaceutical sciences	3	3.1
PharmD^b^	13	13.3
MPharm^c^	25	25.5
Experienced working with PharmD graduates		
Yes	75	76.5
No	23	23.5
Years at current experiences		
1–5 years	31	31.6
6–10 years	30	30.6
11–15 years	20	20.4
16–20 years	10	10.2
>20 years	7	7.2
Type of hospital workplace		
MoPH^d^ sub-district health promoting hospitals	2	2.1
MoPH^d^ community hospitals	50	51.0
MoPH^d^ general/regional hospitals	30	30.6
MoPH^d^ other institutes	4	4.1
Other Ministries	7	7.1
Private hospitals	5	5.1
Setting region		
Bangkok	9	9.1
Central exclude Bangkok	28	28.6
East	3	3.1
North East	29	29.6
North	14	14.3
South	15	15.3
Area of practice^e^		
Consumer protection	11	11.2
Family pharmacist service	21	21.5
Hospital service	66	67.3
Training preceptors	46	46.9

^a^*BPharm* Bachelor of Pharmacy, ^b^*PharmD* Doctor of Pharmacy, ^c^*MPharm* Master of Pharmacy, ^d^*MoPH* the Ministry of Public Health

^e^Hospital pharmacists in Thailand have to get involved in various type of roles and responsibilities which are consumer protection (health promotion and disease prevention, health consumer surveillance), family pharmacist service/home health care and hospital service (drug dispensing, drug purchasing and inventory control, pharmaceutical care service)

**Table 2:** Respondents’ perceptions towards suitability of the PharmD graduates for working in hospital settings and competency differences between the BPharm and PharmD graduates (*n* = 98)

	No. of respondent (%)
Suitability of PharmD graduates for working in hospital settings	
Yes, PharmD graduates are suit for hospital setting.	75 (76.5)
No, PharmD graduates are not suit for hospital setting.	23 (23.5)
Competency differences between BPharm and PharmD graduates	
Yes, there had been the differences.	54 (55.1)
No, there did not have the differences.	44 (44.9)

A chi-squared test or Fisher’s exact test was used to describe the relationship between the respondents’ characteristics (e.g., gender, age group, highest academic degree, work experiences) with their perceptions of the suitability of the PharmD graduates for working in hospital settings.

Experience working with PharmD graduates shows a significant association (*p* < 0.05) with respondents’ perceptions toward the suitability of PharmD graduates for working in hospital settings. Experience working with PharmD graduates and highest academic degree show a statistically significant association (*p* < 0.05) with respondents’ perceptions toward the competency differences between BPharm and PharmD graduates (Table 3). There were no significant interactions among the variables.

**Table 3:** Univariate analyses of relationships between respondents’ characteristic (*n* = 98) and their perceptions, namely suitability of PharmD graduates for working in hospital settings and perceived competency differences between BPharm and PharmD graduates

	Suitability of PharmD graduates for working in hospital settings				Perceived competency differences between BPharm and PharmD graduates			
	Number (%) of respondents		Univariate^a^		Number (%) of respondents		Univariate^a^	
Characteristics	Yes	No	*χ*^2^ value	p-value	Yes	No	*χ*^2^ value	p-value
Gender								
Male	20 (76.9)	6 (23.1)	0.003	0.956	15 (57.7)	11 (42.3)	0.096	0.757
Female	55 (76.4)	17 (23.6)			39 (54.2)	33 (45.8)		
Age range								
<25–35 years	48 (75.0)	16 (25.0)	0.241	0.624	33 (51.6)	31 (48.4)	0.934	0.334
>35 years	27 (79.4)	7 (20.6)			21 (61.8)	13 (38.2)		
Highest academic degree								
PharmD	12 (92.3)	1 (7.7)		0.289^c^	12 (92.3)	1 (7.7)	8.386	0.004^b^
Others (BPharm and MPharm)	63 (74.1)	22 (25.9)			42 (49.4)	43 (50.6)		
Experienced working with PharmD graduates								
Yes	66 (88)	9 (12)	23.405	<0.001^b^	46 (61.3)	29 (38.7)	5.016	0.025^b^
No	9 (39.1)	14 (60.9)			8 (34.8)	15 (65.2)		
Years at current experiences								
0–10	44 (72.1)	17 (27.9)	1.741	0.187	30 (49.2)	31 (50.8)	2.290	0.130
11–20	31 (83.8)	6 (16.2)			24 (64.9)	13 (35.1)		
Work place								
Public	71 (76.3)	22 (23.7)		1.000^c^	51 (54.8)	42 (45.2)		1.000^c^
Private	4 (80.0)	1 (20.0)			3 (60.0)	2 (40.0)		
Setting region								
BKK & central	29 (78.4)	8 (21.6)	0.113	0.737	19 (51.4)	18 (48.6)	0.338	0.561
Other	46 (75.4)	15 (24.6)			35 (57.4)	26 (42.6)		
Area of practice^d^								
Hospital service	51 (77.3)	15 (22.7)	0.062	0.803	36 (54.5)	30 (45.5)	0.025	0.874
Home health care service/family pharmacist & consumer protection	24 (75.0)	8 (25.0)			18 (56.3)	14 (43.8)		
Training preceptor								
Yes	33 (71.7)	13 (28.3)	0.701	0.402	27 (58.7)	19 (41.3)	0.416	0.519
No	38 (79.2)	10 (20.8)			25 (52.1)	23 (47.9)		

^a^Chi-square test was used to determine the association between variables, and Fisher’s exact test was used when more than 20 % of the cells had an expected count of less than 5

^b^Significant at *p* < 0.05

^c^Fisher’s exact test

^d^Responsibilities of Thai hospital pharmacists were including hospital service and also home health care service/family pharmacist & customer protection

Five major themes emerged in response to the open-ended written answers from the survey questionnaire, as presented below.

### PharmD graduates are well suited for patient care services in hospitals

Hospital pharmacists perceived PharmD graduates as being suitable for patient care services in public and private hospitals. They were needed and suitable for the job market in Thailand. Some respondents perceived that the Thai hospital pharmacy job market needs more professionals working in clinical pharmacy services and pharmaceutical care and with multi-disciplinary teams.

*“They have a high quality of academic knowledge, which is suitable for working with other health care teams.” (MPharm (Master of Pharmacy), general/regional hospital).*

*“They are suitable and necessary because they have a role in patient care in hospitals.” (BPharm, general/regional hospital).*

*“The job market needs more clinical pharmacy services and pharmaceutical care.” (PharmD, general/regional hospital).*

Some respondents thought that PharmD graduates were suitable for tertiary care and for employment in large hospitals. This view might be due to the long-standing tradition of providing pharmaceutical care services in tertiary care hospitals, where the hospital pharmacists might have broader experience with medical conditions, treatment and working in a multidisciplinary environment.

*“PharmD pharmacists are suitable for hospitals with sixty beds or more. They can fully show their potential. They will have various cases and have an opportunity to work with multidisciplinary teams.”* (PharmD, community hospital).

In contrast, pharmacists also reported that PharmD graduates were suitable for small hospitals (e.g., sub-district health promoting hospitals and community hospitals) and private hospitals because their role is solely directed toward patient care activities.

*“Yes, suitable. I am working at the MOPH sub-district hospital, which has pharmaceutical care, so, we need to make contact with a multi-disciplinary team.” (PharmD, sub-district health promoting hospital).*

*“PharmD graduates are suitable in community hospitals, where they are involved in patient care.” (BPharm, community hospital).*

*“PharmD graduates are suitable for working in private hospitals”. (BPharm, private hospital).*

### Characteristics of PharmD graduates: high competency and readiness to work

The hospital pharmacists explained the reasons supporting the suitability of PharmD graduates for working in hospitals. Approximately half of the respondents stated that PharmD graduates were suitable for working in hospitals. There was a perception that PharmD graduates had better knowledge and were highly skilled in delivering patient care within the hospital system. They were highly competence for working in the wards and had high efficiency in the clinical pharmacy area.

*“Graduates of the six-year programme have high competency to work on the ward.” (BPharm, community hospital).*

*“They know about the hospital system and are full of knowledge. They can work well.” (BPharm, general/regional hospital).*

Some respondents thought that an important proficiency that supported the PharmD graduates in working in hospitals and providing better patient care were their skills to communicate and coordinate with the wider healthcare team.

*“PharmD graduates have more skills in patient care and communicate with the healthcare team.” (MPharm, community hospital).*

The influences that affected the competency of PharmD graduates might come from the PharmD curriculum. The respondents thought that the PharmD curriculum encouraged students to be exposed to real patient care and provided more clinical knowledge and experiences, as well as holistic care and understanding about patients, than did the BPharm curriculum.

The other strength of PharmD graduates, according to the respondents, was that PharmD graduates had more clerkship experience (e.g., clerkship rotations, clerkship periods and number of mentors). Thus, they had more skills, experiences and expertise and were better at decision making than the BPharm graduates.

Moreover, PharmD graduates were ready to start pharmacy practice efficiently and properly when they first entered the workplace, whereas BPharm graduates needed time to learn to practice and develop clinical skills in the working environment.

*“PharmD graduates learn from real practice, so they are ready to work. But five-year BPharm graduates have to learn before they start their jobs.” (BPharm, community hospital).*

*“The PharmD curriculum emphasises real practice. The students have real experiences before they graduate. Therefore, when they graduate, they can instantly work.” (MPharm, other Ministry).*

Some respondents thought that PharmD and BPharm graduates did not necessarily have significant competency differences because the competency levels might depend on the ability of each individual.

*“No difference. Everyone can learn from working experiences. It depends on the individual.” (MPharm, community hospital).*

Some non-PharmD respondents perceived that the competency of both pharmacy qualifications were similar because they performed similar job activities, particularly standard pharmacy services (e.g., dispensing, procurement and inventory management), which were the first priorities of hospital pharmacy services.

### PharmD graduates need more preparation to work in primary care hospital settings

Respondents thought that PharmD graduates might not be suitable or would be overqualified to work in community hospitals or primary care hospital settings because they perceived that PharmD graduates were trained to work in specialised areas or have to take responsibility for pharmacy duties, which called for “quality work”, for example, in clinical pharmacy activities, pharmaceutical care activities, in-patient department (IPD) services, special clinic services (e.g., HIV clinics), and ward rounds, which are mainly provided in large hospitals. Respondents also perceived PharmD graduates as not being well prepared for working in primary care hospital settings in terms of health promotion and disease prevention.

*“They are suitable in hospital pharmacy work, which emphasise quality work, such as clinical pharmacy activities and pharmaceutical care activities.” (PharmD, general/regional hospital).*

*“PharmD graduates are not suitable in primary care because they are not prepared for trends in the health care system, which emphasise promotion and disease prevention. Therefore, their understanding of clinical pharmacy or other in-depth knowledge will be less used.” (PharmD, community hospital).*

Many respondents were concerned that the community hospital also had other limitations for PharmD graduates to providing pharmaceutical care, such as a limited medicine supply. Most critically ill patients were referred to secondary or tertiary care hospitals. PharmD graduates might be frustrated by their limited role or if they were limited in applying their knowledge or had non-existent opportunities to use the pharmaceutical care skills they had acquired. In-depth knowledge might be less used and thus less useful. This situation might discourage them.

*“They might not be suitable for community hospitals because community hospitals have a limited supply of medicines, medical instruments and other potential. Therefore, these hospitals have limited resources for highly skill patient care.” (BPharm, community hospital).*

*“PharmD graduates are suitable for a large hospital. But in a community hospital, I think it is too much. This pharmacist will be limited by the capacity of a community hospital. They might be discouraged when they work in a community hospital.” (PharmD, community hospital).*

The respondents perceived that PharmD graduates might need more knowledge and skills regarding consumer health protection, health promotion and disease prevention. They suggested that the PharmD curriculum should include more public health subjects and prepare students for primary care roles so that they can work effectively in the community.

*“The six-year PharmD curriculum should have a public health curriculum. PharmD graduates will understand their role and context of work.” (PharmD, MoPH other institute).*

*“A six-year PharmD programme does not teach the current trends for multidisciplinary personnel in a community, such as health promotion and disease prevention. It should not emphasise only pharmacists’ role in a hospital job. This programme missed out on social concerns. They should understand their role and context of work.” (BPharm, community hospital).*

Some respondents suggested that the number of community hospitals and sub-district health-promoting hospitals is higher than tertiary care hospitals. Therefore, faculties might plan to produce PharmD graduates for primary care services as well as tertiary care services and might consider the proportion of PharmD production for each level of care by the number and level of job market needs.

*“PharmD graduates are suitable for about 30–40 % of all hospitals because there are not many general and regional hospitals. There are many community hospitals and sub-district health-promoting hospitals, but they do not have considerable clinical specialty.” (BPharm, Other Ministry).*

### Some PharmD students need to further develop their humanistic skills

Developing PharmD students’ values, attitudes and concerns, as well as knowledge and skills, represents the basic elements to producing pharmacists. However, the most important part of this template is their attitudes, values, ethics and behaviour. The respondents also mentioned an issue with PharmD graduates’ attitudes. Faculties should introduce general and pharmacy-focused professional ethics to students. They should realise their ethical responsibility to uphold their professional values in society.

*“Now, some students have less ethics and respect. Some of them do not respect the preceptor sites; they dress inappropriately, neglect greetings and have less social manners. They should be more enthusiastic for learning. However, they have a good academic knowledge, better than in the past.” (BPharm, community hospital).*

### Unrecognised roles and hospital preparedness for PharmD graduates

Respondents were concerned that some PharmD graduates might struggle to start new services due to the system not encouraging the new role of pharmaceutical care and that PharmD graduates also had to work mainly in dispensing or other non-clinical activities due to the high workload in dispensing services and the shortage of hospital pharmacists. They have had to work at length to implement the new roles, particularly in places where there have been no clinical pharmacists before. They must encourage themselves to move away from traditional dispensing services to new territories, similar to being a clinical pharmacist on a medical ward or working with a health care team.

*“The job market in Thailand does not support or recognise the role of PharmD pharmacists. Therefore, PharmD pharmacists cannot use their potential efficiently.” (BPharm, community hospital).*

Due to the unpreparedness of hospitals to absorb them and with insufficient support plans for PharmD graduates, some of the hospital pharmacists were concerned about the low number of jobs available in the government hospitals for PharmD graduates.

*“There are a lot of pharmacy graduates, but we do not have enough jobs. Why does the government not have jobs available for them? Where is our value?” (BPharm, community hospital).*

Forty-one respondents (41.8 %) thought that the PharmD graduates’ salaries were fair. In some hospitals, PharmD graduates’ salaries were less than other health professionals who also had a 6-year degree, and in some places, the salary was the same as those who had completed a five-year programme.

Two respondents were concerned about the difference in status between temporary employees and government officers or civil servants. They stated that the majority of the new pharmacy graduates were temporary employees and not government officers, and they perceived that temporary employees had less job security and fewer social benefits compare to the government officers. This might discourage their commitment, responsibilities and performance.

*(Note: Thai pharmacy graduates have the option to work in either the public or private sector. Salaries in the public sector are lower than in the private sector for comparable positions; however, the public sector offers other benefits, for example, job security and welfare benefits* [36]*. However, due to the shortage of pharmacists in the public sector, in 1984, the government launched the compulsory working for newly pharmacy graduates in order to recruit the pharmacy graduates from public universities to work in the public sector for two years* [37]*. These graduates had positions as government officers or civil servants, which have several compensations and rewards, for example, current rewards (e.g., base salary, allowances of housing, cost-of-living), future expectation rewards (e.g., pension) and intangible rewards (e.g., job security, social privileges, reputation)* [38–40]*. However, due to the economic crisis and the government's policy of downsizing the public workforce, the compulsory working policy was ended in 1998. Many hospitals faced with a pharmacy workforce shortage problem, so they hire pharmacy graduates as temporary employees but they are often unable to retain these graduates due to the temporary positions provide no opportunities for career advancement* [6]*. In 2006, the compulsory programme was restarted and provides approximately 350* [6] *government officers positions each year while the number of pharmacy graduate is approximately 1,900 per year. The remaining pharmacy graduates may work in the public sector as temporary employees or work in the private sector).*

One was concerned that there has been insufficient preparation for the arrival of PharmD graduates into the workforce, such as a career path in terms of salary and promotion.

*“PharmD is not suitable for my work place. There have not made any preparation for the PharmD graduates. They have salaries and promotions equivalent to five-year BPharm graduates despite the fact that they graduated from a 6-year programme, the same as a physician or dentist. Therefore, they felt upset and discouraged and then quit this job.” (BPharm, Other Ministry).*

## Discussion

The characteristics of the respondents (e.g., most of them are female, age < 35 years, with BPharm degrees and less than 10 years of working experience) are consistent with the previous national pharmacists’ survey [26, 41], except that the previous studies did not include PharmD respondents, as there were only 90 and 128 PharmD graduates in 2006 and 2008, respectively.

Overall, the respondents have positive perceptions of the PharmD graduates. They thought that PharmD graduates were suitable for pharmaceutical care services in hospitals, worked well with health care teams and had thorough knowledge of the hospital system from their clerkship experiences. The results showed a relationship between having experience working with PharmD graduates and the perceptions of the suitability of PharmD graduates for working in hospital settings. Respondents who had experience working with PharmD graduates perceived PharmD graduates as suitable for working in hospital settings because the PharmD graduates were able to collaborate with other health care professionals [42] and were able to work as clinical pharmacy practitioners and clinical services, such as the in-patient department (IPD) services, special clinic services (e.g., HIV clinics), and ward rounds, which are mainly provided in large hospitals [19].

Responses also pertained to aspects of care regarding the types of hospitals, which consists of primary care for less acute illnesses and focuses on health promotion/prevention of diseases, secondary care for chronic cases, and tertiary care for severe cases that require hospitalisation [43, 44]. Some respondents noted that PharmD graduates might not be suitable for primary care hospital settings because the respondents perceived that the majority of pharmaceutical care as taking place in tertiary care settings. They were also concerned about PharmD graduates’ competency for providing primary care services. The finding in this study is in line with a previous study in which PharmD graduates reported their competency in consumer health protection, which is mainly activity in primary care hospital settings, as being lower than PSc-BPharm graduates [6]. This situation is similar to other countries in which the curriculum provided inadequate public health subjects in the PharmD programme [45]. Pharmacy education should include and integrate the subject of health promotion and public health. Competency in health promotion is one of the recommendations of the FIP*Ed* Global Competency Framework and in other countries [46–48].

There are 9,976 primary care settings, 774 secondary care settings and 89 tertiary care settings in Thailand. Pharmacy workforce needs indicated in the 2012–2016 National Health Service Plan at each level of care are as follows: Primary care needs one pharmacist per 10,000 people (pharmacy workforce from hospital), secondary care needs 2–12 pharmacists/setting, and tertiary care needs 9–58 pharmacists/setting [44]. The proportion of pharmacists in tertiary care compared to secondary care and primary care should be approximately 35 and 65 %, respectively. Therefore, faculties should consider producing more general PharmD graduates for primary care and secondary care settings who work for standard pharmacy services (e.g., procurement and inventory management, pharmaceutical compounding, out-patient and in-patient services, pharmaceutical individual care, and community pharmaceutical care, such as home care, primary health care, public health community service and consumer protection). Faculties should continue to produce specialised PharmD graduates (e.g., cardiovascular, oncology, trauma and neonatal) for tertiary care. However, few faculties planned to produce PharmD graduates to provide primary and secondary care.

There was also a relationship between two characteristics (e.g., experience working with PharmD graduates and highest academic degree) and the perception of competency differences between the PharmD and BPharm graduates (*p* < 0.05).

The majority of PharmD respondents, some non-PharmD respondents and the majority of the respondents who have experience working with PharmD graduates perceived the competency differences between two pharmacy qualifications. Possible reasons for these trends are as follows:

1. The PharmD graduates had perceived higher competencies (more clinical knowledge, more clinical skills, better decision making) than BPharm graduates, particularly in pharmaceutical care activities. This finding was similar to the findings from the study about employer satisfaction that the PharmD graduates had high skills in providing pharmaceutical care, had self-learning competencies and had high responsibility in their work [19].The competency in clinical problem solving and decision making in patient care are also noted in the competency standards of many countries (e.g., Thailand, the US, Canada, and Australia) [14, 42, 47, 48].
2. PharmD graduates were ready, upon qualification, to work clinically, whereas BPharm graduates needed time to learn to practice. This observation was consistent with reports about employer and colleague satisfaction (e.g., pharmacists, nurses, and physicians) of PharmD graduates from one faculty of pharmacy in Thailand that PharmD graduates had the appropriate competencies and were ready to work [19]. They only needed a short period of induction [49]. One Thai pharmacy faculty, and an article in the publication of the white paper “Pharmacy in England: building on strengths, delivering the future”, also set the aim of ensuring that their PharmD graduates are ready to work from day one to deliver quality services to patients and populations [50–52].

Moreover, Thai patients reported a difference between PharmD and non-PharmD graduates in that they provided better medicine and health information and had an excellent service mind [19].

Some non-PharmD respondents thought that the competency of both pharmacy qualifications were similar because they performed similar job activities, particularly standard pharmacy services or non-clinical activities (e.g., dispensing, procurement and inventory management), which were the top priorities of hospital pharmacy services. This might be because hospital pharmacists experienced a high dispensing workload and a shortage of pharmacy staff. In some hospitals, pharmacists have had to manage those basic activities; otherwise, they would not be able to provide special clinical activities (e.g., ward rounds). Respondents also noted that in some hospitals, PharmD graduates have struggled to implement clinical roles, particularly in places where there had been no clinical pharmacists before. They must position themselves to move away from traditional dispensing services to working as clinical pharmacists on a medical ward or working with a health care team.

Similar findings were reported in the US during the 1980s and 1992, i.e., that BPharm and PharmD graduates had similar job activities (e.g., prescription processing, patient care) and spent a similar amount of time in those activities. PharmD graduates have mainly worked as nonclinical skilled staff rather than in clinical positions, possibly due to a higher level of drug distribution responsibilities [53–55]. In addition, some respondents explained that competencies were individualistic and that BPharm graduates will increase their competencies by practicing. Thus, if BPharm graduates acquired more experiences and continued practicing in pharmaceutical care activities, they would have the same competency as PharmD graduates.

Respondents were also concerned about the development of PharmD graduates’ roles in patient care; these concerns are consistent with several studies that noted barriers to providing pharmaceutical care services in hospitals. Examples of such impediments include lack of time, high dispensing workload, shortage of pharmacy staff, lack of recognition of the pharmacy professional by other health care providers, lack of funds or a low financial incentive, and lack of employer’s recognition about the pharmacist’s role and responsibilities [26, 56–59].

Many of the respondents were concerned about the limited number of jobs available from the government for PharmD graduates. Jobs were limited due to financial constraints, even though there is predicted to be a shortage of hospital pharmacists until 2019 [3]. However, career opportunities in private settings appear abundant and therefore present a more optimistic situation [3, 60]. World-class private hospitals, which provide high-quality services (e.g., hospital accreditation in Thailand, International Organization for Standardization (ISO), Joint Commission International), have a high demand for PharmD graduates to provide specialist pharmaceutical care. The need for PharmD graduates in local private hospitals and local drug stores still requires further study.

Some respondents were concerned about the communication skills and attitudes of the students. These are the same concerns as those of the global independent commission on the Education of Health Professionals for the 21^st^ Century [61]. Health professionals’ education should develop their ethical conduct as well as providing and mobilising their knowledge [62].

The respondents also had concerns about the healthcare system’s preparedness for PharmD graduates (e.g., lack of adequate salary, compensation and career path). This finding is consistent with those of Thammatacharee et al. [63], who found imbalances in the financial circumstances of healthcare professionals. New medical and dental graduates receive higher salaries if they are working in rural areas, whereas pharmacists do not. The lack of maintenance factors, such as proper strategies by policy makers and hospital administration committees toward professional career development (e.g., performance and competency evaluation), might also affect pharmacists’ motivation [64].

Stakeholders should increase their awareness about the preparedness for this new generation of pharmacists for the challenges they will inevitably meet. The following recommendations emerged from the findings.

The PharmD curriculum should include more public health subjects and prepare students for health promotion and health education roles to meet local pharmacy practice needs as well as global pharmacy competency and service standards [65, 66]. Encouraging professional ethical issue in curriculum is also important as well as the knowledge and skills to be a successful pharmacist [67, 68].

Academic institutes should collaborate in planning and designing the curriculum to produce pharmacy graduates by closely following the national service plan that stipulates the need to increase the pharmacy workforce in tertiary care compared to secondary care and primary care by approximately 35 and 65 %, respectively.

Professional bodies and the government should clearly define job descriptions, career structures and career development for PharmD graduates [69, 70].

This study has some limitations. First, the respondents in this study were recruited by using convenience sampling. The effort was devoted to identifying hospital pharmacists. However, the lists we developed were not complete due to the absence of an up-to-date database representing the numbers and addresses of hospital pharmacists [3, 26]. Second, the response rate was quite low at 55.6 %. Generalization might need to be addressed. Third, the number of years that the respondents worked among BPharm and PharmD graduates was not included in the questionnaire even though this factor might influence the respondents’ perceptions. Fourth, the questionnaire used dichotomous questions (yes/no), which had some advantages, such as being time-efficient, thus simplifying data coding and analysis. However, the open-ended answers showed that the respondents could provide more than a yes/no response. For example, some respondents answered that the suitability of PharmD graduates in hospitals depends on the type of hospital setting. Therefore, exploring perceptions might not be as simple as yes or no. Further study might consider other types of question (e.g., multiple-choice questions or Likert scales) for the several possible answers, which would allow respondents to express their true uncertainty concerning their perceptions [71, 72].

Finally, the survey did not provide a detail of eight domains of competency standard guidelines required for both BPharm and PharmD graduates for the licensure examination during the 2002–2014 period and did not contain the specific question about the eight domains of competency that the participants perceived differences between BPharm and PharmD graduates. This study also did not measure competency objectively. Further research should specify the competency domain in which the hospital pharmacists’ perceived differences between the graduates of those two programmes and should also investigate the PharmD graduates’ actual competency [6].

Researchers with both Thai and non-Thai pharmacy backgrounds participated in the data analysis to minimise any bias in the findings [35, 73, 74]. To establish the quality of the analysis of qualitative data of the research, the criteria for “trustworthiness” are described as follows:

- *Credibility:* this study did not present the analysis to the participants because the information from the questionnaire was anonymous. However, triangulation [32] was used to confirm the results with the researchers’ team, whose members are hospital pharmacists, as well as by employing documentary evidence.
- *Transferability*: this study might serve as an overview for educators in other developing countries who might wish to consider the implications of this study.
- *Dependability*: the methodological description was reported to allow this study to be repeated.
- *Confirmability*: the researchers’ bias was reduced by using triangulation, and several researchers were involved in the development and cross-checking of the themes to ensure that the findings were the perceptions of respondents rather than the researchers [69, 75].

## Conclusions

PharmD graduates were suited for tertiary care and for employment in large hospital settings, as they were well coordinated with the health care team and were trained to work in specialised areas (e.g., pharmaceutical care activities, specialised clinic services and ward rounds). However, there were concerns about the suitability of the PharmD graduates for community hospital/primary care hospital settings due to the insufficiency of health promotion and disease prevention training in the curriculum. Approximately half of the respondents perceived a competency difference between the two pharmacy qualifications. They thought that the PharmD graduates had higher competencies (e.g., more clinical knowledge, more clinical skills, better decision making) than BPharm graduates, particularly in pharmaceutical care activities, and were prepared to work, whereas BPharm graduates needed time to learn to practice clinically. The other half of the respondents perceived the competency of both pharmacy qualifications as being similar because they had similar job activities, particularly in non-clinical activities, where hospital pharmacists had a high dispensing workload and there was a shortage of pharmacy staff. Thus, PharmD graduates had to work in non-clinical activities, which indicated that both degrees had the same competency. PharmD graduates have struggled to implement a clinical role. They need an encouraging environment and must inspire themselves to move away from traditional dispensing services to work as clinical pharmacists on a medical ward or within a health care team.

## Additional files

Additional file 1: **Differences among the curriculum structures of the 5-year BPharm, the 2008 Announced 6-year PharmD programme and the 2012 Announced 6-year PharmD programme [**9**,** 12**,** 13**].** (DOCX 31 kb) Additional file 2: **Differences among the competency standard guidelines for licensure examination of the 5-year BPharm, the 2008 Announced 6-year PharmD programme and the 2012 Announced 6-year PharmD programme [**14**–**17**].** (DOCX 29 kb)

## Abbreviations

FIP*Ed*: International Pharmaceutical Federation Education Development Team
IP-PD: Industrial Pharmacy-PharmD programme
MoPH: Ministry of Public Health
MPharm: Master of Pharmacy
PC-BPharm: Pharmaceutical Care track-BPharm programme
PC-PD: Pharmaceutical Care-PharmD programme
PCT: The Pharmacy Council of Thailand
PECT: The Pharmacy Education Consortium of Thailand
PharmD: Doctor of pharmacy
PhD: Doctor of Philosophy
PSc-BPharm: Pharmaceutical Sciences track- BPharm programme
SAP: Social and Administrative Pharmacy
UNESCO: The United Nations Educational, Scientific and Cultural Organisation
US: The United States of America
WHO: The World Health Organization
