# Supplementary material for: Hospital pharmacists’ perceptions of the suitability of doctor of pharmacy graduates in hospital settings in Thailand
Source: BMC Med Educ. 2015 Oct 24;15:181. doi: 10.1186/s12909-015-0471-6 (PMC4619343; doi:10.1186/s12909-015-0471-6)
Supplement: Additional file 1: — Differences among the curriculum structures of the 5-year BPharm, the 2008 Announced 6-year PharmD programme and the 2012 Announced 6-year PharmD programme [9, 12, 13]. (DOCX 31 kb) [file 12909_2015_471_MOESM1_ESM.docx]

**Additional file 1:**

Differences among the curriculum structures of the 5-year BPharm, the 2008 Announced 6-year PharmD programme and the 2012 Announced 6-year PharmD programme.

|  | The 5-year BPharm programme^a^ | The 2008 Announced  6-year PharmD programme^b^ | The 2012 Announced  6-year PharmD programme^b^ |
| --- | --- | --- | --- |
| Characteristics | Separated into various tracks/subspecialties, for example,  -Clinical pharmacy  -Pharmaceutical technology  -Social Pharmacy/ Social and Administrative Pharmacy (SAP)  -Research and development | Focused in pharmaceutical care | Divided into 2 main tracks: Pharmaceutical care PharmD (PC-PD) programme and industrial pharmacy PharmD (IP-PD) or pharmaceutical sciences programme |
| Curriculum structure | Credits (minimum) | Credits (minimum) | Credits (minimum) |
| 1. General education (e.g., computers, humanities, language and communication, sciences and mathematics, statistics) | 30 | 30 | 30 |
| 2. Pharmacy courses | 120 | 144 | 144 |
| 2.1 Basic sciences (e.g., anatomy, biochemistry, microbiology, physiology) |  | 30 | 30 |
| 2.2 Professional courses (e.g., pharmaceutical technology, pharmacotherapy, pharmacy administration, pharmacy orientation, forensic pharmacy, senior project) |  | 114  **-Pharmaceutical care area:** not less than 42 credits or 30% of pharmacy course  **-Pharmaceutical sciences area:** not less than 35 credits or 25% of pharmacy course  **- Social and Administrative Pharmacy area:** not less than 14 credits or 10% of pharmacy course  **-Specialty professional or special track**: N/A | 114  **-Pharmaceutical care area:** not less than 30 credits or 25% of pharmacy course  **-Pharmaceutical sciences area:** not less than 30 credits or 25% of pharmacy course  **- Social and Administrative Pharmacy area:** not less than 15 credits or 12% of pharmacy course  **-Specialty professional or special track:** not less than 45 credits or not less than 37% of the pharmacy course |
| 2.3 Professional practice | 500 hours | 2,000 hours^c^  **-Clerkship in core competencies:** 400 hours  **-Clerkship in specialised area:** 1,600 hours | 2,000 hours^c^  **-Clerkship in core competencies:** 400 hours  **-Clerkship in specialised area:** 1,600 hours |
| 3. Free elective courses | 3 | 6 | 6 |
| Total credits required | 150-188 | 220 | 220 |

^a^ The 5-year BPharm and structure follows curriculum standard for higher education.

^b^ The 2008 Announced 6-year PharmD curriculum and the 2012 Announced 6-year PharmD curriculum structure follows Thailand Qualification Framework (TQF) for certification of Doctor of Pharmacy degree and the Pharmacy Council of Thailand

^c^ One professional practice credit represents between 45 and 60 hours of practice training
