# Supplementary material for: Hospital pharmacists’ perceptions of the suitability of doctor of pharmacy graduates in hospital settings in Thailand
Source: BMC Med Educ. 2015 Oct 24;15:181. doi: 10.1186/s12909-015-0471-6 (PMC4619343; doi:10.1186/s12909-015-0471-6)
Supplement: Additional file 2: — Differences among the competency standard guidelines for licensure examination of the 5-year BPharm, the 2008 Announced 6-year PharmD programme and the 2012 Announced 6-year PharmD programme [14–17]. (DOCX 29 kb) [file 12909_2015_471_MOESM2_ESM.docx]

**Additional file 2:**

Differences among the competency standard guidelines for licensure examination for the 5-year BPharm, the 2008 Announced 6-year PharmD programme and the 2012 Announced 6-year PharmD programme.

|  | The 5-year BPharm programme | The 2008 Announced 6-year PharmD programme | The 2012 Announced 6-year PharmD programme^b^ |
| --- | --- | --- | --- |
| Competency-oriented content in programme  (Patient-:Product-:SAP-oriented content) | 35%:49%:16% | 44%:33%:23% | 40%:40%:20% |
| Competency standard guidelines of the Pharmacy Council of Thailand | -The competency standards (2002) contained the following 8 domains;  1. Pharmaceutical manufacturing process and quality assurance  2. Management of pharmacy resources  3. Preparation of pharmaceutical products  4. Delivery of primary health care and health promotion  5. Contribution to the optimum use of medicines  6. Provision of medicine management  7. Provision of medicine information  8. Legal practice | | -There are three Thai pharmacy competency guidelines (e.g., core competency guidelines, functional competency standards for pharmaceutical care and industrial pharmacy).  **-The core competency standards** (2012) contained the following 7 domains;  1. Professional ethics and morals  2. Working with team and system management  3. Information technology skills, communication skills and providing education on medicine usage.  4. Pharmaceutical products, herbal medicinal products, pharmaceutical chemical products and quality control  5. Drug procurement and extemporaneous preparation to patients  6. Pharmaceutical care and herbal use  7. Health system and health care system  **-A functional competency standard in pharmaceutical care** (2011) contained the following 9 domains;  1. Integrate knowledge in pharmaceutical manufacutring and quality assurance process in pharmaceutical care services  2. Provide drug selection and drug procurement of pharmaceutical products to serve individual patient and community appropriately  3. Prepare appropriate pharmaceutical products for individual customer  4. Provide advice or conduct activities for individual and community health promotion  5. Understand the pharmacy professional-related laws  6. Provide pharmaceutical care services for individual patient and community as a part of health care team  7. Manage medication system and provide medication risk management for patient safety with health care team  8. Manage medicine system quality assurance to maintain the effectiveness and safety of patient care  9. Provide evidence-based, accurate, current, and reliable medicines and health-related product information to individual patient, community and health care providers.  **-A functional competency standard in industrial pharmacy** (2014) contained the following 4 domains;  1. Research and development  2. Quality assurance and quality control  3. Manufacturing  4. Regulatory affairs and product registration |
| Pharmacy licensure examination | - All pharmacy graduates are required to pass the same licensure examination regardless of different competencies acquired from any pharmacy programme. | | - The pharmacy students who started their pharmacy education in and after 2015 will have two licensure examinations.  -First examination for core competencies at the end of the 4^th^ year  -Second examination for specialized area at the end of the 6^th^ year |
|  | - There has been one pharmacy license provided in Thailand.  - Pharmacy graduates who obtain the pharmacy licence are able to work in any of the pharmacy practice areas independent of their specialty. | | |
